# Calmodulin Gene Expression in Response to Mechanical Wounding and *Botrytis* *cinerea* Infection in Tomato Fruit

**DOI:** 10.3390/plants3030427

**Published:** 2014-08-29

**Authors:** Hui Peng, Tianbao Yang, Wayne M. Jurick

**Affiliations:** 1 Food Quality Laboratory, Beltsville Agricultural Research Center, Agricultural Research Service, United States Department of Agriculture, Beltsville, MD 20705, USA; E-Mails: hui.peng@ars.usda.gov (H.P.); wayne.jurick@ars.usda.gov (W.M.J.); 2 College of Life Sciences, Guangxi Normal University, Guilin 541004, China

**Keywords:** calcium signaling, plant defense, salicylic acid, jasmonic acid, postharvest decay

## Abstract

Calmodulin, a ubiquitous calcium sensor, plays an important role in decoding stress-triggered intracellular calcium changes and regulates the functions of numerous target proteins involved in various plant physiological responses. To determine the functions of calmodulin in fleshy fruit, expression studies were performed on a family of six calmodulin genes (*SlCaMs*) in mature-green stage tomato fruit in response to mechanical injury and *Botrytis cinerea* infection. Both wounding and pathogen inoculation triggered expression of all those genes, with *SlCaM2* being the most responsive one to both treatments. Furthermore, all calmodulin genes were upregulated by salicylic acid and methyl jasmonate, two signaling molecules involved in plant immunity. In addition to *SlCaM2*, *SlCaM1* was highly responsive to salicylic acid and methyl jasmonate. However, *SlCaM2* exhibited a more rapid and stronger response than *SlCaM1*. Overexpression of *SlCaM2* in tomato fruit enhanced resistance to *Botrytis*-induced decay, whereas reducing its expression resulted in increased lesion development. These results indicate that calmodulin is a positive regulator of plant defense in fruit by activating defense pathways including salicylate- and jasmonate-signaling pathways, and *SlCaM2* is the major calmodulin gene responsible for this event.

## 1. Introduction

Calcium is a universal second messenger involved in growth, development and mediating responses to a variety of abiotic and biotic stresses in plants [1,2,3]. Cellular changes in calcium are captured by calcium sensors containing the EF-hand motif. Calmodulin (CaM) is a ubiquitous calcium sensor in plants, and plays an important role in almost all aspects of cell activity [4,5,6]. In contrast to animals which have one or a few *CaM* genes encoding identical isoforms, plants have multiple genes encoding more diversified isoforms. In all plants examined, *CaM* genes, even genes encoding the same isoform, are differentially expressed in response to numerous external stimuli such as touch, heat shock, cold, light, pathogens, and to phytohormones. In *Arabidopsis*, seven *CaM* genes encode four highly conserved isoforms. A loss-of-function mutant in *Arabidopsis* *AtCaM2* affects pollen germination [7]. The *atcam3* knockout mutant exhibits reduced thermo-tolerance after heat treatment, whereas overexpression of *AtCaM3* significantly increases thermo-tolerance [8]. In soybean, specific CaM isoforms *SCaM-4* and *SCaM-5*, are highly induced either by a fungal elicitor or pathogen attack, whereas three other *SCaMs* show no response to these stimuli [9]. Transgenic tobacco plants overexpressing *SCaM-4* and *SCaM-5* display spontaneous lesions and constitutive expression of systemic acquired resistance-associated genes. Thus, the level of individual CaM proteins is differentially regulated in plants upon exposure to various stimuli. 

Tomato (*Solanum lycopersicum* L.) is the second most important vegetable crop, and the total worldwide production in 2012 was 161.8 million tons with a farm gate value of $80 billion. However, over 25% of fresh produce including tomato fruit are lost after harvest due to the mechanical damage during handling, transportation, and decay [10]. Previous studies on the function of tomato *CaMs* have focused on their role in vegetative tissues. Bergey and Ryan (1999) reported an accumulation of *CaM* mRNA and CaM protein in tomato leaves after wounding or systemin treatment, suggesting that it plays a role in plant defense [11]. Zhao* et al.* (2013) reported that there are six *CaM* genes in tomato encoding for four isoforms [12]. SlCaMs in leaves were highly responsive to a variety of biotic and abiotic stimuli. Silencing SlCaM2 and SlCaM6 altered expression of defense-related genes and reduced resistance to pathogens. However, there is a gap in the literature concerning the importance of CaM in the ripening of fleshy fruit, as well as in response to stresses that can be encountered during postharvest handling and storage. In this study, we report an expression analysis of *CaM* gene family in response to postharvest stresses, and characterization of the functional significance of a specific *CaM* gene responsible for disease resistance in tomato fruit.

## 2. Results

### 2.1. SlCaMs Are Responsive to Mechanical Wounding

**Figure 1:**
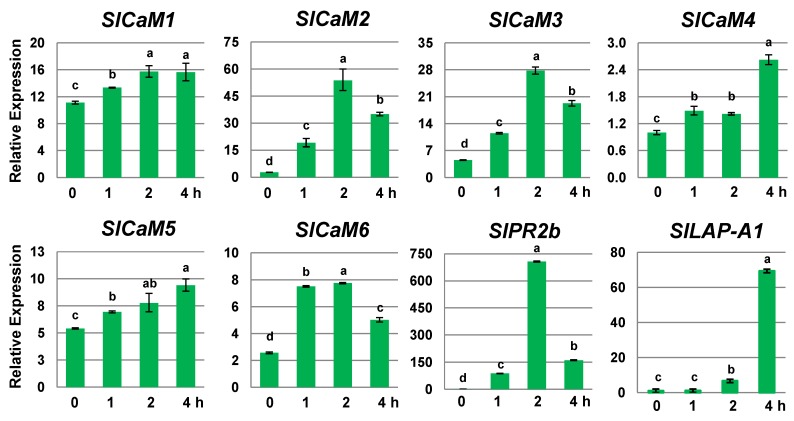
Expression of *SlCaMs* in wounded tomato fruit. Transcription levels of *SlCaMs* were measured by RT-qPCR. Relative gene expression levels are shown following normalization with actin transcript values. Error bars represent standard error of the mean. For each gene, different letters indicate statistically significant differences among mean values (*p* < 0.05). The results are based on at least three repeats in three independent experiments.

To study the effect of wounding on *SlCaMs* expression, mature green stage fruit were selected because this is the specific stage that is routinely harvested by the tomato industry, Without wounding, the most abundantly expressed genes were *SlCaM1* and *SlCaM5* (Figure 1). After wounding, the expression of all *SlCaM*s were stimulated within one hour after treatment and peaked following 2–4 h. Among them, *SlCaM2* showed the most profound stimulation. Its expression increased more than 10-fold in one hour, and peaked at 32-fold within two hours. Wounding also increased the expression of *SlCaM3*, *SlCaM4* and *SlCaM6* 2–4 fold, albeit at lower levels. However, wounding had little effect on the expression of *SlCaM1* and *SlCaM5*. *SlPR2b* and *SlLAP-A1*, two genes known to be induced by wounding [13,14] were used as a positive control, and their expression was triggered by wounding as expected. These results collectively suggest that *SlCaMs* in tomato fruit are wound-responsive.

### 2.2. Pathogen Infection Triggers Calmodulin Gene Expression

**Figure 2:**
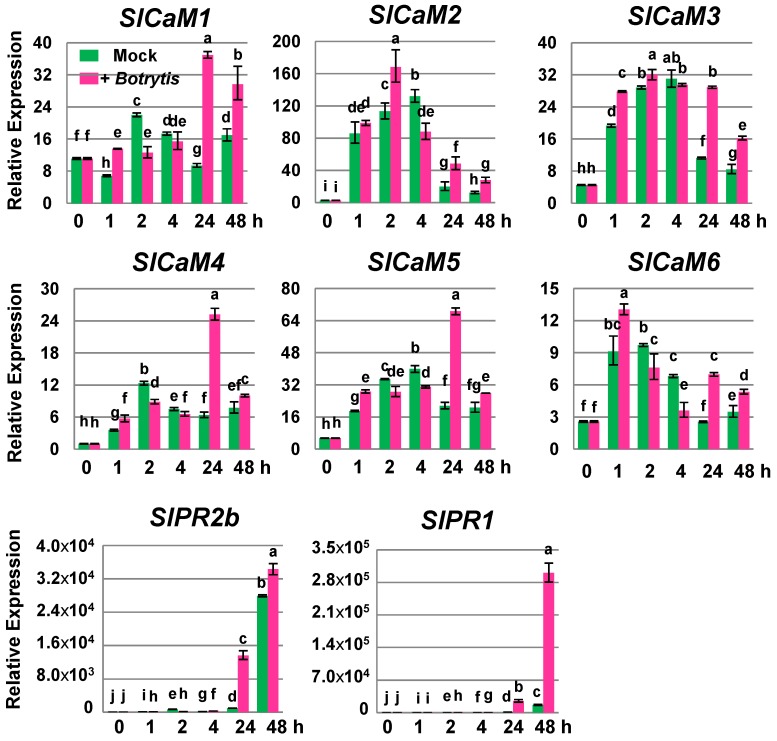
Expression of *SlCaMs* in response to fungal infection. Tomato fruit at the mature-green stage were mechanically wounded and immediately treated with water (mock inoculation) or inoculated with *Botrytis cinerea* conidia*.* The wounded and wounded-plus-inoculated areas were excised after 0 to 48 h of incubation at 20 °C. Total RNA samples for RT-PCR were isolated from pericarp tissue and transcript levels of *SlCaMs* genes were determined by RT-qPCR. Relative gene expression levels are shown following normalization with actin transcript values. For each gene, different letters indicate statistically significant differences among mean values (*p* < 0.05). The results are based on at least three repeats in three independent experiments.

Wounding is a prerequisite for most fungal postharvest pathogens that cause decay during storage. To investigate the effects of pathogen infection on *SlCaM*s expression, wounded tomato fruit were inoculated with the necrotrophic fungal pathogen, *Botrytis cinerea.* The growth of the fungus was observed two days postinoculation. The inoculated wounds displayed extensive water soaking, mycelial growth, and necrosis. In contrast, the control treatments (mock inoculation), wounds containing water only, were asymptomatic. Additionally, the expression levels of *SlCaM*s in response to pathogen infection were investigated. Since wounding triggered *SlCaM*s gene expression, much of the increased *SlCaM*s in early time points after both mock and pathogen-inoculation may have resulted from wounding (Figure 2). However, the pathogen-treatment had a more profound and prolonged stimulation of all *SlCaMs* than the mock inoculation. After 24 h of treatment, expression differences between pathogen- and mock inoculation were more distinguishable. For instance, *SlCaM2* exhibited the most dramatic stimulation in response to pathogen infection and mock inoculation immediately after treatment, suggesting that this stimulatory effect resulted mainly from physical wounding. However, following 24 h inoculation, the wound effect on *SlCaM2* expression declined, and the pathogen effect was evident. Similarly, pathogen treatment enhanced the expression of *SlCaM1*, *SlCaM3*, *SlCaM4* and *SlCaM5* 24 h post inoculation. *SlPR2b* was stimulated by 87–106 fold after 1 h of treatments mainly due to the effect of wounding. However, it was increased over 13,600 fold at 24 h post inoculation. Interestingly, the expression of *SlPR1* was remarkably high after 24 h post inoculation. These results suggest that *SlCaMs* could be involved in signaling events during fungal infection by *B. cinerea*. 

### 2.3. Calmodulin Genes Are Salicylic Acid-Responsive

**Figure 3:**
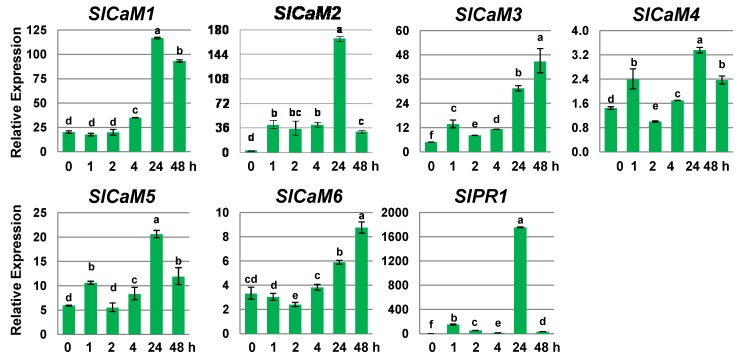
*SlCaMs* expression levels in response to salicylic acid treatment. Transcription levels of *SlCaMs* genes were investigated by quantitative real time RT-qPCR. Mature green stage fruit were treated with 4 mM salicylic acid for different time periods as indicated. Relative gene expression levels are shown following normalization with actin transcript values. Error bars represent the standard error of the mean. For each gene, different letters indicate statistically significant differences among the means (*p* < 0.05). The results are based on at least three repeats in three independent experiments.

Salicylic acid (SA) is a key signaling molecule for the activation of genes involved in systemic acquired resistance to both biotic and abiotic stresses [13,15]. Treating harvested fruits with SA can help reduce decay incidence by activating defense genes such as *PR-1* and *PR-2* [16,17,18,19,20]. To determine whether SA affects the expression of *SlCaMs*, fruits were treated with salicylic acid for different time periods ranging from 0 to 48 h. Previously, it was observed that *SlPR1*, a marker of SA-regulated gene expression, showed the highest induction at 4 mM [21], and thus this concentration of SA was utilized in the following experiments. All *SlCaMs* were upregulated by SA (Figure 3). There were two peaks for the expression of *SlCaM3*,* SlCaM4* and *SlCaM5*. The first peak (minor) occurred one hour after treatment, and second peak (major) appeared at or after 24 h. This pattern was similar to that of the SA-responsive gene *SlPR1*. In comparison, only one peak was observed for *SlCaM1* and *SlCaM6* after 8 or 24 h of treatment. However, the highest induction was observed after 24 h of treatment for all CaMs.

### 2.4. Methyl Jasmonate Stimulates Calmodulin Gene Expression

**Figure 4:**
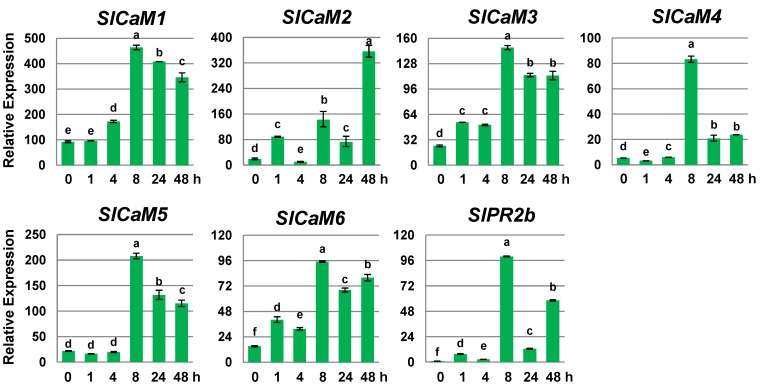
*SlCaMs* gene expression in response to methyl jasmonate treatment. Transcription levels of *SlCaMs* genes were investigated by quantitative real time RT-qPCR. Mature green stage fruit were treated with 20 μM methyl jasmonate for different periods of time. Relative gene expression levels are shown following normalization with actin transcript values. Error bars represent standard error of the mean. For each gene, different letters indicate statistically significant differences among mean values (*p* < 0.05). The results are based on at least three repeats in three independent experiments.

Jasmonic acid (JA) is another signal molecule that regulates plant responses to wounding/insect stress and necrotrophic pathogen attack [13,22]. To investigate the expression patterns of *SlCaMs* in response to JA, fruits were treated with 20 μM methyl jasmonate (MeJA), the methyl ester of JA. The expression of all *SlCaMs* increased after applying MeJA (Figure 4). The stimulatory response for *SlCaM2*, *SlCaM3* and *SlCaM6* was detected one hour after treatment. However, significant changes were evident 4 h post treatment for *SlCaM1*, and at 8 h post treatment for *SlCaM3*, *SlCaM4* and *SlCaM5*. The most JA-responsive genes were *SlCaM1* and *SlCaM2*. It is interesting to note that the expression of *SlCaM2* exhibited a wave-like pattern in response to MeJA. The first peak appeared at one hour, the second at 8 h, and the third at 48 h, which was similar to the expression pattern of the JA-responsive gene *SlPR2b*. These results suggest that *SlCaM2* is both a JA early responsive and late responsive gene. Taken together, *SlCaMs* positively respond to JA in tomato fruit, which is opposite to JA’s effect on those genes in leaf tissue. Interestingly, the expression of all six *SlCaMs* in tomato leaves was inhibited by MeJA treatment, suggesting that there is tissue differential expression for *SlCaMs*’ response to JA [12].

### 2.5. Overexpression of the SlCaM2 Gene Reduces Symptoms Incited by Botrytis Cinerea

**Figure 5:**
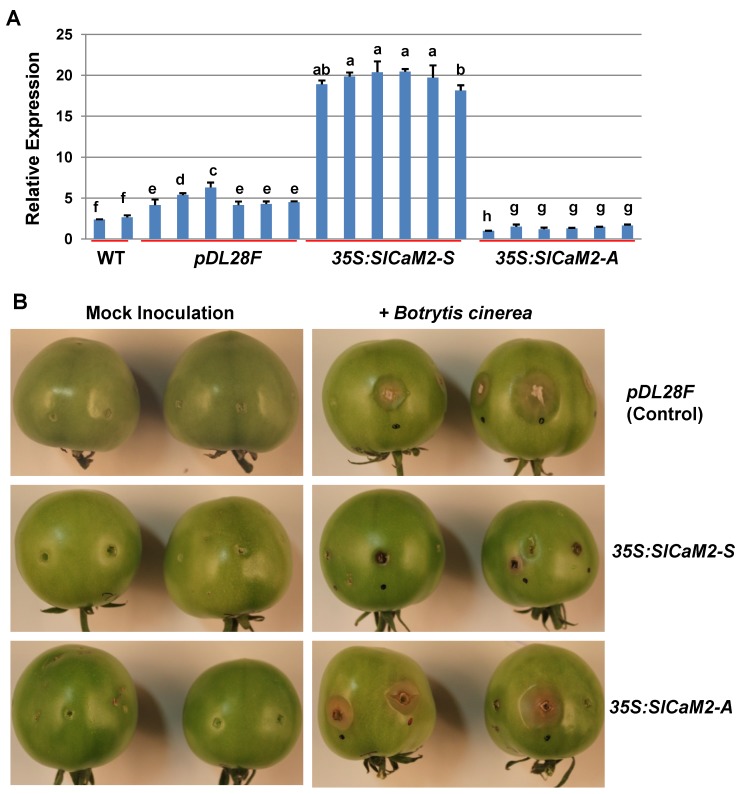
Expression level of *SlCaM2* in tomato fruit in relation to *B. cinerea* infection. Mature green stage fruits were agroinjected with *A. tumefaciens* carrying different constructs. *pDF28F*, empty vector (control); *35S:SlCaM2-S*, pDL28F carrying *SlCaM2* in the sense orientation; *35S:SlCaM2-A*, pDL28F carrying *SlCaM2* in the antisense orientation. (**A**) Examination of expression levels of *SlCaM2* in different transgenic fruits. 48 h after agroinjection, a piece of pericarp tissue from each fruit was used for RT-qPCR. The results are based on at least three repeats in three independent experiments. For each gene, different letters indicate statistically significant differences among mean values (*p* < 0.05). (**B**) Pathogen resistance and susceptibility test shows that overexpression of *SlCaM2* enhanced resistance to *B. cinerea*. The fruits agroinjected with different constructs were inoculated into a wound with water (mock) or *B. cinerea* conidia, and put in a moisture saturated container to observe decay development. The photos were taken 72 h post inoculation.

Since *SlCaM2* was the only gene highly responsive to wounding and pathogen infection, as well as SA and MeJA, it was selected for further functional analysis *in planta*. To quickly assess transgene expression in fruit, we selected the agroinjection method to transiently express *SlCaM2* in the early mature-green stage tomato fruit. We introduced *SlCaM2*-sense and antisense constructs into tomato fruit via *Agrobacterium tumefaciens*. Forty-eight hours after injection, the expression level of *SlCaM2* in control (vector alone) was ~2–3 fold higher than non-transgenic fruit (WT) (Figure 5A), suggesting that *Agrobacterium* itself could stimulate the expression of the endogenous *SlCaM2* expression. However, as compared to empty vector alone, the expression level of *SlCaM2* in the sense fruit was over five fold higher 48 h after injection. In contrast, the expression in the antisense fruit was reduced 4–5 times as compared to the vector only control. These results demonstrate that the transient *Agrobacterium*-mediated transformation methodology was a viable choice to study candidate gene function in tomato fruits.

At 48 h post agroinjection, *B. cinerea* conidial suspensions or water were pipetted into wounds (via nail punctures) on transgenic fruit, and monitored for decay development. In the first 24 h, no obvious symptoms were observed. At 2 days postinoculation, control and antisense fruit started to display water soaking and necrosis, while the pathogen inoculated wounds in sense fruit showed some slight necrosis. Three days postinoculation, the control fruit exhibited severe decay, as lesion development was evident at the inoculation site which included mycelial proliferation, accompanied by soft watery circular lesions that were delineated by healthy tissue. The antisense fruit had similar symptoms of decay compared to the control (Figure 5B). However, sense fruit showed limited necrosis and limited water-soaking. All the mock inoculated fruit were asymptomatic at the inoculation sites. These results indicate that increased expression of *SlCaM2* enhanced resistance to *B. cinerea*, and ultimately limited fungal growth, lesion development, and colonization in tomato fruit.

## 3. Discussion

Fleshy fruits are an important part of the human diet, providing fiber, minerals, nutrients and various other substances beneficial to human health. Nearly a quarter of all fresh fruits and vegetables in the U.S. market are lost after harvest due to damage caused by abiotic and biotic stresses such as wounding and pathogen infection [10]. The importance of calcium in fruit ripening and postharvest handling has been recognized for many years. Calcium is most frequently associated with delayed ripening maintaining fruit quality (particularly firmness), and preventing decay through the strengthening of cell walls by cross-linking de-esterified pectic acid residues [23,24,25,26,27,28,29,30]. However, it is well-recognized that calcium mediated signaling is important for plant responses to a wide variety of stresses including mechanical touch and pathogen attack [1,2,3,31]. Intracellular calcium changes in response to those stimuli are perceived and decoded by calcium sensors including CaM [4,5,6]. Further, *CaM* genes themselves respond to the biotic and abiotic signals. In tomato, wounding or systemin increased the accumulation of *CaM* in leaves [11]. Zhao* et al.* (2013) reported that SlCaMs in tomato leaves were highly responsive to a variety of biotic and abiotic stimuli [12]. Silencing of *SlCaM2* and *SlCaM6* led to the reduced resistance to tobacco rattle virus and *Pythium aphanidermatum*. However, it did not affect the resistance to *Pseudomonas syringae* and *Xanthomonas oryzae*. In this study, we found that six *SlCaMs*, especially *SlCaM2*, in tomato fruits were upregulated by mechanical wounding, necrotrophic fungal infection, SA and JA. It is important to note that *SlCaMs* genes respond to JA in leaves and fruits very differently. Thus, there may be tissue specificities for *SlCaMs* expression in response to stresses that accompany functional changes in different tissues.

SA and JA are two major signal molecules that mediate plant defense responses. In general, the SA-dependent pathway is activated by biotrophic pathogens, while the JA pathway is triggered by necrotrophs and herbivore attack/wounding [13,22,32,33]. The interactions between those two pathways can be antagonistic or synergistic [34,35]. It has been reported that the necrotrophic pathogens such as *Botrytis cinerea* can produce an exopolysaccharide, which acts as an elicitor of SA pathway in tomato [36,37]. We observed that SA-responsive gene *SlPR1*was remarkably stimulated by *Botrytis* infection which supported their findings (Figure 2). It has been well documented that the synthesis of SA and SA signaling are under extensive regulation by calcium signaling [38,39,40,41,42]. Cross-talk between the JA signaling pathway and calcium signaling also occurs [43,44]. A calcium/CaM-regulated transcription factor AtSR1/CAMTA3 has been shown to be involved in both SA- and JA-dependent pathways [45,46]. Analysis of the AtSR1/CAMTA3 gene knockout mutant showed enhanced resistance to both biotrophic and necrotrophic pathogens [41,47]. Previously, the expression patterns of *SlSRs*, the orthologs of *AtSRs/CaMTAs* in tomato, were analyzed. All of them displayed a positive response to both SA and MeJA in tomato fruit [21]. Therefore *SlSRs* could be candidate targets for *SlCaMs* in controlling disease resistance. In addition, there are quite a few other CaM-target proteins involved in SA-signaling that respond both positively and negatively [38,39,40,41,42]. Considering six *SlCaM* genes encoding four isoforms, the regulation of wounding and disease resistance in tomato fruit by calcium/CaM is hypothesized to be complex. Nevertheless, *SlCaM2* will be a first choice for further expression and functional analysis studies in tomato fruits. Introducing *SlCaM2* into tomato, by classical breeding or through molecular means, might enhance the tolerance/resistance to mechanical injury, pathogens, and other stresses leading to the availability of high quality fresh fruit for consumption.

## 4. Experimental Section

### 4.1. Plant Materials 

Tomato plants (*S. lycopersicum* cv. Moneymaker) were grown in a greenhouse at 28 °C with a 16 h/8 h (light/dark) cycle. Fruit were harvested at the mature green stage (MG), as defined by USDA-ARS criteria [48], when the fruits were physiologically mature but not yet ripe. In the industry, tomatoes are often harvested at this stage for packing and shipment, and subsequently treated with ethylene to promote ripening prior to sale. The greenhouse-grown MG fruit were held under ambient conditions overnight to reduce harvest shock prior to treatment.

### 4.2. Mechanical Injury, Methyl Jasmonate and Salicylic Acid Treatments

Wounding was executed by manually cutting fruit pericarp into one inch pieces with a sharp knife at room temperature. The fruit pieces were put in a plastic box with the wet towel to maintain high humidity for the indicated time period. For SA treatment, fruits were immersed in 4 mM SA solution for 0 to 48 h. For MeJA treatment, fruits were sealed in a jar with 20 μM MeJA. After each treatment, pericarp samples were immediately frozen in liquid nitrogen and stored at −80 °C.

### 4.3. Pathogen Infection and Decay Assay

Fruits were mechanically injured and infected by inoculation with a conidial suspension of the fungal pathogen *B. cinerea* strain 22B which was isolated from naturally infected apple fruit [49]. The fungus was propagated via single spore isolate and maintained on Potato Dextrose Agar. The inoculation was done essentially as described previously [21]. Briefly, fruit were punctured (3 mm depth, 2 mm diameter) at six sites around the equator of each fruit; 3 sites with 10 µL of conidial suspension (1 × 10^4^ conidia/mL), and the other three with 10 µL of sterile Tween20-treated water for mock inoculation. After inoculation, the fruit were stored in plastic sealed containers with moist towels to maintain high humidity and kept at 20 °C. Pericarp tissue samples were obtained from inoculated fruits by using a cork borer to isolate the tissue immediately surrounding the inoculated area at different intervals after treatment. The pericarp tissue was collected from fruit at the different time points, frozen in liquid nitrogen and stored at −80 °C.

### 4.4. RNA Extraction and RT-qPCR

Total RNA was isolated from frozen tissue using RNeasy Plant Mini Kit following the manufacturer’s instruction (Qiagen, Valencia, CA, USA). Reverse transcription and qPCR were performed as described [21]. Briefly, one μg of total RNA was used to synthesize cDNA with iScript^™^ kit (Bio-RAD, Hercules, CA, USA), RT-qPCR analysis of cDNA was performed on a CFX96 Real-time System (Bio-RAD). Gene specific primers listed in Table 1 were designed with the Primer3 software [50]. The efficiency coefficient E was calculated for all primer pairs individually by plotting the relationship between Cq value (threshold cycle) and log[cDNA]. All reactions were performed in triplicate from three independent samples. Cq was used for relative quantification of the input target number. Relative fold difference (*N*) was the number of the treated target gene copies relative to the untreated control gene copies and is calculated as follows: *N* = 2^ΔCq^. ΔCq was the difference in threshold cycles for *SlCaMs* targets and the *actin* internal reference. Relative gene expression (fold changes) was calculated based on *N* with the lowest value as 1. Student’s *t* test (*p* < 0.05) was used to determine the significant difference of relative expression of individual genes among different treatments and controls (Microsoft Excel 2007).

**Table 1:** Primers used for qPCR and cloning.

Primer Name	Oligonucleotides	Gene ID
SlCaM1-a	CCAGAGTTCCTTAACCTGATGG	Solyc01g008950
SlCaM1-b	CTTTTCGCCTAGGTTTGTCATC	
SlCaM2-a	TCTGAGGAGGAGTTGAAAGAGG	Solyc10g081170
SlCaM2-b	TCAACATCAGCTTCCCTAATCA	
SlCaM3-a	GATGGTAATGGAACCATCGACT	Solyc10g077010
SlCaM3-b	CATCAGTGAGCTTCTCACCAAG	
SlCaM4-a	TCAGATCTCGGAGTTCAAAGAAG	Solyc11g072240
SlCaM4-b	CAGGTTAAGGAACTCAGGGAAGT	
SlCaM5-a	TTAACTTGATGGCTCGGAAGAT	Solyc12g099990
SlCaM5-b	ACGAATCATCTCGTCAACCTCT	
SlCaM6-a	ATCACTTGGTCAGAATCCCACT	Solyc03g098050
SlCaM6-b	AGCTGCAGAAATAAAGCCATTC	
SlPR1-a	CTGTGAAGATGTGGGTTGATGAG	NM-001247429
SlPR1-b	TCTCCAGTTACCTGGTGGATCAT	
SlPR2b-a	TCTTGCCCCATTTCAAGTTC	M80608
SlPR2b-b	TGCACGTGTATCCCTCAAAA	
SlLAPA1-a	TGTCGCAGCATGTGAAAATATG	Solyc12g010020
SlLAPA1-b	AGCACCAGTTAATGTTGCCAGA	
SlActin-a	GAAATAGCATAAGATGGCAGACG	X55749
SlActin-b	ATACCCACCATCACACCAGTAT	
SlCaM2-S1 *	ggtggtaccATGGCGGATCAGCTGACGG	
SlCaM2-S2 *	ggaggatccCTTGGCCATCATGACCTTAAC	
SlCaM2-A1 *	ggtggtaccCTTGGCCATCATGACCTTAAC	
SlCaM2-A2 *	ggaggatccATGGCGGATCAGCTGACGG	

* Primers used for cloning full length *SlCaM2*. The underlined portion indicates the restriction enzyme site.

### 4.5. Construction of Ti Plasmids Carrying Sense- and Antisense-SlCaM2 Gene

The full length tomato *SlCaM2* was amplified from a mixture of fruit tissues by Pfx DNA polymerase, and subcloned to TA cloning Kit (Life Technology, Grand Island, NY, USA) using gene specific primers (Table 1). The nucleotide sequences of the positive clones were confirmed by sequencing. The full length of *SlCaM2* was subcloned into pDL28F, a derivative of pCambia1300 [41] in the sense- and antisense- orientations in the sites of *Kpn* I and *Bam*H I downstream of 35S promoter, and introduced into *Agrobacterium tumefaciens* strain GV3101. The positive clones were verified by PCR using gene-specific primers.

### 4.6. Fruit Agroinjection

Agroinjection of tomato fruit was carried as described [51]. Briefly, agrobacterium cultures were grown overnight from individual colonies at 28 °C in YEB medium plus selective antibiotics, transferred to induction medium (0.5% beef extract, 0.1% yeast extract, 0.5% peptone, 0.5% sucrose, 2 mM MgSO_4_, 20 μM acetosyringone, 10 mM MES, pH 5.6) plus antibiotics, and grown again overnight. The next day, cultures were resuspended with infiltration medium (10 mM MgCl_2_, 10 mM MES, 200 μM acetosyringone, pH 5.6 with OD_600_ of 1.0), and incubated at room temperature with gentle agitation (20 rpm) for a minimum of 2 h. Cultures were injected into the early mature green fruit using a syringe with a 0.5- × 16-mm needle. The needle was introduced into the fruit tissue through the stylar apex and blossom end. Because of differences in fruit size, the injection was terminated when the solution started running off the injection site.

## 5. Conclusions

Calmodulin, a ubiquitous calcium receptor in the eukaryotic cell, plays an important role in almost all aspects of cell activity in plants [4,5,6]. Previous studies have suggested that calmodulins in tomato vegetative tissues respond to a variety of biotic and abiotic stimuli, such as wounding or systemin treatment [11] and pathogen attack [12]. We have observed that the expression levels of six *SlCaMs* in mature green tomato fruit are stimulated by mechanical injury and *B. cinerea* infection. Among the six genes, *SlCaM2* was the most responsive to both pathogen infection and wounding. Further, expressions of all *SlCaMs* were upregulated by SA and MeJA which occurs 24 and 8 h after treatment, respectively. However, *SlCaM2* also had a detectable peak as early as one hour after treatment by SA and MeJA. In general, *SlCaMs* in tomato fruit are early wound-responsive genes, but late responsive genes to pathogen attack, JA and SA. *SlCaMs* may regulate the wound response and disease resistance via SA- and/or JA-dependent signaling in fruit. Based on its gene expression level, *SlCaM2* is the major *CaM* gene in tomato fruit that responds to mechanical injury and pathogen attack. Transient expression of *SlCaM2* into mature green tomato fruit significantly increases resistance to *B*. *cinerea.* Conversely, reducing its expression facilitates pathogen growth in the host. Since *SlCaM2* is an SA-responsive gene, it will be interesting to test whether increasing its expression also confers resistance to biotrophic pathogens (*i.e.*, *Cladosporium fulvum*). Moreover, it will be of great utility to identify target(s) of *SlCaM2* to improve overall fruit quality by strengthening tolerance to wounding and concomitantly increasing disease resistance to fungal postharvest plant pathogens.
